# Molecularly Engineered Wing‐Shaped Azobenzene Memristors for Logic‐in‐Memory and Edge Visual Intelligence

**DOI:** 10.1002/advs.76309

**Published:** 2026-06-26

**Authors:** Yanze Liu, Tao Han, Jiahui Ding, Hong Lian, Lingling Yao, Zhaoxin Xu, Xingyu Zhang, Shuanglong Wang, Jiangnan Xia, Tianchi Zhang, Weiwei Kang, Qingchen Dong

**Affiliations:** ^1^ MOE Key Laboratory of Advanced Display and System Applications Shanghai University Shanghai P. R. China; ^2^ School of Mechanical & Electronic Engineering and Automation Shanghai University Shanghai P. R. China; ^3^ Microelectronics and Optoelectronics Technology Key Laboratory of Hunan Higher Education School of Physics and Electronic Electrical Engineering Xiangnan University Chenzhou P. R. China; ^4^ Department of Applied Physics The Hong Kong Polytechnic University Hong Kong SAR P. R. China; ^5^ Third Hospital of Shanxi Medical University Shanxi Bethune Hospital, Shanxi Academy of Medical Sciences Tongji Shanxi Hospital Taiyuan P. R. China

**Keywords:** azobenzene derivatives, charge transfer, logic gates, neuromorphic computing, organic resistive random access memory

## Abstract

Organic molecular resistive memory offers a promising platform to overcome the von Neumann bottleneck. Here, we report four symmetric azobenzene‐based small molecules with diverse terminal substituents (nitroimidazole, imidazole, carbazole, and triphenylamine) for memristive applications. By tuning the terminal groups, the devices exhibit tunable nonvolatile behaviors—ranging from ternary/binary WORM to bipolar nonvolatile resistive memory—all featuring high ON/OFF ratios, low operating voltages, and excellent stability. Mechanistic studies reveal that charge‐transfer‐induced conformational changes govern the twisted intramolecular charge‐transfer states, dictating these distinct memory characteristics. Notably, the Cz‐methylene‐Azo memristor demonstrates continuous conductance tunability and essential synaptic functions (e.g., excitatory postsynaptic current (EPSC), paired‐pulse facilitation (PPF), long‐term potentiation/depression (LTP/D)). Furthermore, it serves as a versatile logic‐in‐memory unit, executing multiple logic gates (OR, AND, XOR, NAND, etc.), the half and full‐adder circuits. Its applicability for in‐memory computing is successfully validated via convolutional neural networks (CNN)‐based image edge detection, highlighting its great potential for next‐generation integrated organic neuromorphic architectures.

## Graphical abstract

Rational engineering of terminal substituents in symmetric azobenzene‐based molecules enables precise control over conformationally coupled charge‐transfer processes. This design yields tunable nonvolatile resistive memory behaviors, ranging from write‐once‐read‐many‐times (WORM) to rewritable switching. The resulting organic memristors execute versatile logic‐in‐memory operations and synaptic functions, successfully driving convolutional neural networks for advanced neuromorphic computing.

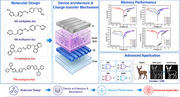

## 1 Introduction

While the era of artificial intelligence (AI) and internet of things (IoT) has inspired visions of comprehensive global connectivity, realizing this future is hindered by the physical limitations of silicon scaling and the von Neumann bottleneck inherent in traditional architectures [1, 2, 3]. To transcend these barriers, it is essential to develop computing paradigms that fundamentally improve data storage density and processing speed while maintaining high energy efficiency [4, 5]. As a result, resistive switching technologies, including resistive random access memory (RRAM), ferroelectric random access memory (FeRAM), phase‐change random access memory (PRAM), magnetoresistive random access memory (MRAM), and memristor‐based synaptic devices, are being extensively investigated as key enablers for the next generation of computing architectures [6].To date, a variety of functional materials have been used in the fabrication of resistive switching memory devices, including organic materials, inorganic materials, and organic‐inorganic hybrid materials. Among them, organic small molecules have attracted considerable attention from researchers due to their low cost, light weight, and excellent scalability, and have been widely applied in the field of resistive memory [7, 8, 9, 10, 11, 12]. Rational molecular design can effectively regulate the storage performance of devices, and donor‐acceptor (D‐A) type molecules represent the simplest and most effective molecular construction strategy, requiring only the connection of different donor and acceptor groups [13, 14, 15].

The azo group has garnered significant attention in photothermal storage and optical switching due to its unique photo‐ and thermoisomerization capabilities [16, 17, 18]. While previous studies in electronic memory have often treated the azo moiety simply as a weak electron‐withdrawing unit, we strategically utilize it as a multifunctional molecular core [19]. By leveraging this core, the systematic terminal functionalization of azo‐based molecules offers an effective strategy to comprehensively modulate resistive switching performance. In 2021, Zhou et al. embedded tetraphenylethylene modified with azobenzene (TPE‐Azo) into flexible poly(ethylene‐co‐maleic anhydride) (PEM), resulting in the TPE‐Azo@PEM composite material. This composite was used as the active layer in FTO/TPE‐Azo@PEM/Ag devices, which exhibited excellent ternary c (WORM) memory behavior. In the same year, Miao et al. developed a conjugated small molecule DAPAPIT with two azo groups, which enabled memory devices to exhibit excellent nonvolatile ternary memory characteristics. The experimental results revealed that, with increasing external bias, charge carriers were gradually injected into two types of charge traps at different depths within the DAPAPIT molecular backbone, resulting in distinct conductive states [19]. Building on these foundations, we recognize that the azo group serves as a moderate electron‐accepting center within a D–A framework. Compared to stronger acceptor units, this moderate nature is essential because it allows the terminal substituents to dominantly modulate the charge‐transfer strength, trap depth, and molecular energy levels. Furthermore, the azobenzene unit is conformationally active. The N═N bond and its adjacent aromatic rings can undergo bias‐induced charge redistribution and torsional deformation, providing a conformation‐coupled charge‐transfer pathway that is crucial for stabilizing or destabilizing the charge‐separated state.

To establish a unified molecular platform and systematically investigate these structure‐property relationships, we utilized the azobenzene core as a common backbone and introduced four distinct terminal groups to construct a systematic molecular series with different electronic effects, charge‐transfer strengths, conformational responses, and charge‐trapping characteristics. Specifically, imidazole (IMI) and nitroimidazole (NIZ) were incorporated as electron‐deficient heterocyclic terminal groups. IMI was selected to provide a weak electron‐withdrawing reference, whereas NIZ introduces an additional nitro group to create deeper trapping sites and stronger electron‐withdrawing ability. This comparison allows us to explicitly evaluate how trap depth and electron‐accepting strength affect the binary/ternary switching behavior. On the other hand, carbazole (Cz) and triphenylamine (TPA) were selected as representative electron‐donating terminal groups with distinct geometric features. Carbazole and triphenylamine are widely utilized as donor units due to their different electronic properties. Carbazole units are prized for their rigid planar structure and strong electron‐donating ability, which facilitate reversible oxidation, excellent hole mobility, and high thermal stability [20, 21]. In contrast, triphenylamine features a propeller‐like nonplanar geometry and a larger aromatic framework. Therefore, the incorporation of Cz and TPA is fundamentally intended to reveal how different donor geometries and conformational changes affect charge‐transfer stabilization, rather than merely comparing their degree of extensive π‐conjugation. While triphenylamine derivatives offer even more extensive π‐conjugation, their performance can be further tailored. Specifically, introducing a methylene bridge disrupts planarity to form non‐coplanar small molecules [22]. This steric configuration effectively suppresses intramolecular charge transfer (CT) in favor of stable intermolecular CT, thereby significantly improving memory switching characteristics [23].

Herein, a series of symmetric azobenzene molecules with different terminal group substitutions (NIZ‐methylene‐Azo, IMI‐methylene‐Azo, Cz‐methylene‐Azo, and TPA‐methylene‐Azo) were synthesized by introducing nitroimidazole, imidazole, carbazole, and triphenylamine groups on both sides of the azobenzene molecular backbone via methylene linkers. These compounds were subsequently employed as active materials for the fabrication of memory devices. The results demonstrate that the memory behaviors are significantly governed by both the electronic effects of the terminal groups and the geometry‐dependent conformationally coupled CT. Memristors based on NIZ‐methylene‐Azo and TPA‐methylene‐Azo exhibit nonvolatile ternary and binary WORM memory behaviors, respectively, while IMI‐methylene‐Azo and Cz‐methylene‐Azo devices show bipolar nonvolatile resistive memory characteristics. All devices exhibit markedly enhanced electrical performance, characterized by high I_ON_/I_OFF_ ratios, low threshold voltages (V_th_), and excellent cycle‐to‐cycle reproducibility. Further experimental and theoretical analyses confirm our design rationale: molecular conformational changes occur following charge transfer, and the presence of a conformationally coupled transfer state introduces distinct energy barriers. This results in different degrees of CT complex dissociation and consequently dictates whether the device exhibits WORM or bipolar nonvolatile resistive memory behavior. Moreover, they demonstrate diverse electrically tunable synaptic functionalities, including multilevel conductance states corresponding to synaptic weights, excitatory postsynaptic current (EPSC), paired‐pulse facilitation (PPF), and long‐term potentiation and depression (LTP/D). Further experimental and theoretical analyses indicate that molecular conformational changes occur following CT, and the presence of a conformationally coupled transfer state introduces distinct energy barriers, resulting in different degrees of CT complex dissociation and consequently distinct memory behaviors. Moreover, the memristors based Cz‐methylene‐Azo served as building blocks for constructing a series of digital logic gates (including OR, AND, NOR, NAND, XOR, and XNOR). To validate the logic computation capability, half‐adder and full‐adder circuits were designed and verified through simulation. The resulting memristive crossbar arrays based Cz‐methylene‐Azo support analog edge enhancement and collaborative multi‐class recognition with convolutional neural networks (CNNs), achieving near‐software accuracy and demonstrating strong potential for high‐efficiency, hardware‐based edge visual intelligence.

## 2 Results and Discussion

**FIGURE 1:**
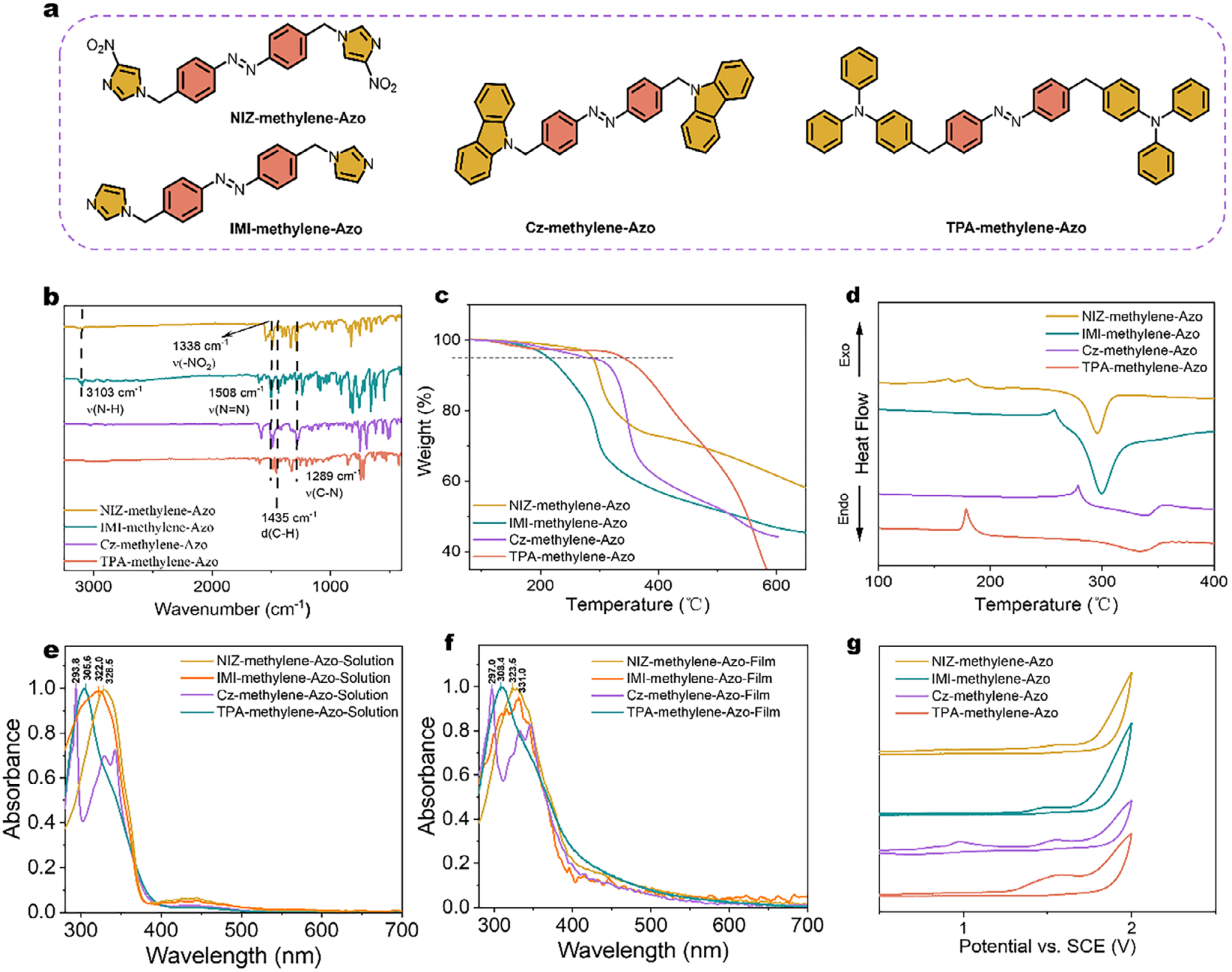
(a) Synthetic routes of NIZ‐methylene‐Azo, IMI‐methylene‐Azo, Cz‐methylene‐Azo, and TPA‐methylene‐Azo. (b) FT‐IR spectra of NIZ‐methylene‐Azo, IMI‐methylene‐Azo, Cz‐methylene‐Azo, and TPA‐methylene‐Azo. (c) TGA curves and (d) DSC thermograms of the four compounds under a nitrogen atmosphere. UV–vis absorption spectra of the compounds in (e) THF solutions and as (f) thin films. (f) Cyclic voltammograms (CVs) of the four compounds.

The detailed synthetic procedures [24] and corresponding chemical structures of the target molecules and their intermediates are outlined in Figure 1a and Scheme S1. The synthesis initiated with the bimolecular reduction of *p*‐nitrotoluene to form Me‐Azo (40% yield), followed by radical bromination to produce the key intermediate Br‐methylene‐Azo (67% yield). NIZ‐methylene‐Azo and IMI‐methylene‐Azo were synthesized in 70% yields via nucleophilic substitution between Br‐methylene‐Azo and the sodium salts of 4‐nitroimidazole and imidazole, respectively. Similarly, Cz‐methylene‐Azo was obtained in 42.6% yield through the nucleophilic attack of the carbazolide anion on the electrophilic methylene carbon of Br‐methylene‐Azo. Finally, the TPA‐methylene‐Azo derivative was realized via Suzuki–Miyaura coupling in 56% yield. Nuclear magnetic resonance (NMR), High‐resolution mass spectrometry (HRMS) and Fourier transform infrared (FT‐IR) spectroscopy were employed to verify the chemical structures of the synthesized compounds. Figures S1–S12 illustrate the ^1^H NMR, ^13^C NMR and HRMS spectra that confirm the chemical compositions of the final molecules. As shown in Figure 1b, characteristic FT‐IR absorption bands were clearly observed for all target molecules. The peak located at 1289 cm^−^^1^ is assigned to C─N bending vibrations, whereas the band at 1435 cm^−^^1^ corresponds to the C─H stretching vibrations of the methylene spacer. The absorption around 1508 cm^−^^1^ is associated with the vibrations of the azo (N═N) linkage. Notably, the absorption at 3103 cm^−^^1^ in NIZ‐methylene‐Azo and IMI‐methylene‐Azo is attributed to the C─H stretching vibrations of the imidazole ring. Furthermore, a distinct peak at 1338 cm^−^^1^ in the spectrum of NIZ‐methylene‐Azo is characteristic of the symmetric stretching vibration of the nitro (─NO_2_) group. Thermogravimetric analysis (TGA) and differential scanning calorimetry (DSC) were employed to evaluate the thermal stability of the four target compounds (NIZ‐methylene‐Azo, IMI‐methylene‐Azo, Cz‐methylene‐Azo, and TPA‐methylene‐Azo), ensuring their thermal robustness under device‐operating condition [25].

As illustrated in Figure 1c,d, the onset decomposition temperatures (T_d_, corresponding to 5% weight loss) for NIZ‐methylene‐Azo, IMI‐methylene‐Azo, Cz‐methylene‐Azo, and TPA‐methylene‐Azo were determined to be 289.1°C, 210.3°C, 284.9°C, and 336.5°C, respectively. Their corresponding melting points (T_m_) were 296.6°C, 299.5°C, 342.8°C, and 337.2°C. Notably, no distinct glass transition temperature (T_g_) was observed for any of the compounds. The broad exothermic peaks observed at 179.8°C, 257.6°C, and 278.3°C for NIZ‐methylene‐Azo, IMI‐methylene‐Azo, and Cz‐methylene‐Azo, respectively, suggest partial crystallization, whereas the sharp exothermic peak at 178.1°C for TPA‐methylene‐Azo indicates complete crystallization. These results demonstrate that all four molecules possess excellent thermal stability. Figure 1e,f presents the UV–vis absorption spectra of all four molecules in THF solution and as thin films. The absorption bands of all compounds in the thin‐film state exhibit a distinct redshift compared to their solution counterparts. In solution, NIZ‐methylene‐Azo and IMI‐methylene‐Azo display two characteristic peaks: an intense band around 330 nm attributed to the *π*→*π* ^*^ transition of the azobenzene moiety, and a weaker band near 450 nm arising from the *n*→*π* ^*^ transition of the N═N bond [26]. For Cz‐methylene‐Azo, the strong peak at 293 nm is assigned to the azobenzene *π*→*π* ^*^ transition. The shoulder peaks observed at 328 and 342 nm originate from the *π*→*π* ^*^ transitions of the carbazole unit, while the absorption in the low‐energy region (420–470 nm) corresponds to the *n*→*π* ^*^ transition. Similarly, TPA‐methylene‐Azo exhibits a major peak at 304 nm (*π*→*π* ^*^) and a low‐energy band between 420–470 nm (*n*→*π* ^*^) [27, 28, 29, 30, 31]. In the thin‐film state, the absorption onset wavelengths (*λ*_onset_) for the NIZ‐, IMI‐, Cz‐, and TPA‐ derivatives were determined to be 529, 587, 541, and 535 nm, respectively. Consequently, the optical bandgaps (E_g_) were calculated to be 2.34, 2.11, 2.31, and 2.29 eV, using the equation *E*_g_ = 1240/*λ*_onset_ [32]. The photophysical properties of all molecules are summarized in Table S1. The highest occupied molecular orbital (HOMO) and lowest unoccupied molecular orbital (LUMO) energy levels were calculated based on UV–vis absorption spectra and cyclic voltammetry (CV) data, as shown in Figure 1g. The HOMO levels for NIZ‐methylene‐Azo, IMI‐methylene‐Azo, Cz‐methylene‐Azo, and TPA‐methylene‐Azo were calculated to be −5.45, −5.51, −5.22, and −5.60 eV, respectively, with corresponding LUMO levels of −3.11, −3.40, −2.93, and −3.29 eV. As summarized in Table S2, the HOMO energy levels generally increased with the enhanced electron‐donating ability of the substituents.

**FIGURE 2:**
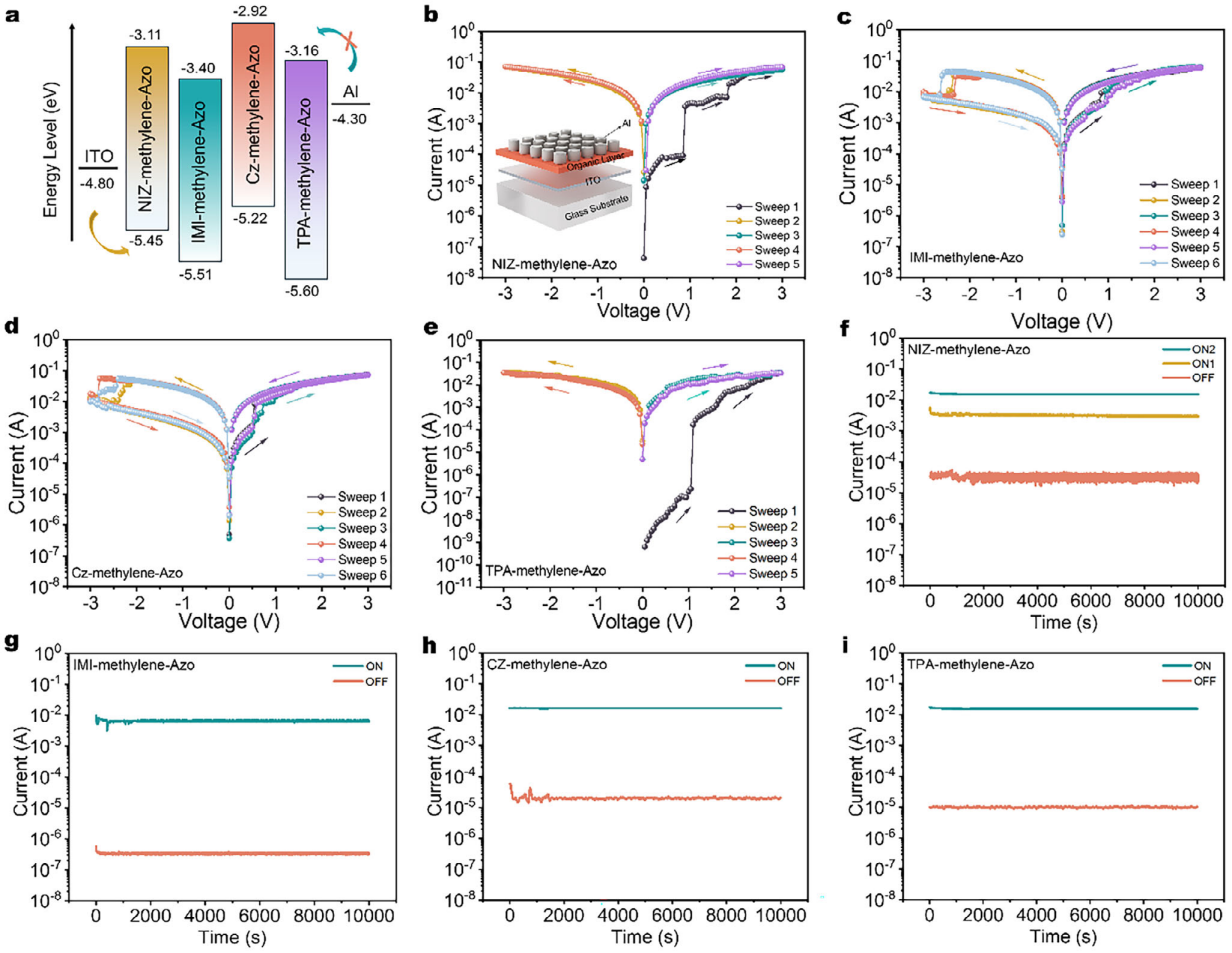
(a) Diagram showing the energy levels within the respective devices. *I*–*V* characteristics of (b) NIZ‐, (c) IMI‐, (d) Cz‐, and (e) TPA‐methylene‐Azo‐based devices (inset: device structure). Retention performance of *I*–*V* characteristics of (f) NIZ‐, (g) IMI‐, (h) Cz‐, and (i) TPA‐methylene‐Azo‐based devices.

The corresponding energy level alignment of the active materials within the devices is illustrated in Figure 2a. Therefore, the introduction of appropriate substituents proved effective in modulating molecular energy levels and bandgaps, thereby potentially enhancing carrier transport efficiency. The current‐voltage (*I*–*V*) characteristics of the memory devices are presented in Figure 2b–e. Figure 2b inset depicts the device architecture, in which the organic layer is interposed between Al and ITO electrodes. The four molecules were fabricated on pre‐patterned ITO glass substrates via spin‐coating. Figure 2b illustrates the I‐V characteristics of the NIZ‐methylene‐Azo‐based device, which exhibits three distinct conductivity states. Initially in the low‐conductivity (OFF or 0) state, the device undergoes two abrupt current transitions at threshold voltages (V_th_) of 0.8 and 1.8 V, respectively, during the first positive sweep (0 to 3 V, Sweep 1). These transitions correspond to a switch from the OFF state to an intermediate state (ON1) and subsequently to a high‐conductivity state (ON2), indicating electrical tristability and serving as the ‘writing’ process. The device retains the ON2 state during the subsequent negative scan (Sweep 2) and remains stable under consecutive biases (Sweeps 3–5). Consequently, the device demonstrates typical ternary WORM characteristics with an I_ON2_:I_ON1_:I_OFF_ current ratio of 10^3^:10^2^:1. Figure 2c presents the I‐V characteristics of the ITO/IMI‐methylene‐Azo/Al device. Despite the structural similarity between IMI‐methylene‐Azo and NIZ‐methylene‐Azo, the former exhibits distinct memory behavior. Initially in a low‐conductivity (OFF or 0) state, the device undergoes a sharp current increase at a V_th_ of 0.8 V during the first positive sweep (0 to 3 V, Sweep 1). This transition to a high‐conductivity (ON or 1) state corresponds to the writing process. Conversely, during the negative sweep (0 to ‐3 V, Sweep 2), the current drops abruptly at −2.32 V, returning the device to the OFF state, which serves as the ‘erasing’ process. This reversible switching behavior is maintained under consecutive biases. Consequently, the device demonstrates electrical bistability and typical bipolar nonvolatile resistive memory characteristics, with an I_ON_/I_OFF_ current ratio of 10:1. Figure 2d illustrates the I‐V characteristics of the Cz‐methylene‐Azo‐based device. During the initial positive sweep from 0 to 3 V (Sweep 1), the device remains in a low‐conductivity state (OFF or 0) until it reaches a V_th_ of 0.5 V. At this point, an abrupt current transition occurs, switching the device to a high‐conductivity state (ON or 1); this corresponds to the ‘writing’ process. The device retains this ON state even under initial reverse bias. However, when the negative bias reaches −2.4 V (Sweep 2), the current drops rapidly from 10^−1^ A to 10^−3^ A, indicating a transition back to the OFF state, which serves as the ‘erasing’ process. Following this erasure, the device can be switched back to the ON state in subsequent cycles. Consequently, the ITO/Cz‐methylene‐Azo/Al device demonstrates bistable resistive switching and typical bipolar nonvolatile resistive memory behavior. The *I*–*V* curves for the ITO/TPA‐methylene‐Azo/Al device are presented in Figure 2e. Initially in a low‐conductivity state (OFF or 0), the device undergoes a sharp current increase at a V_th_ of 1.0 V during the 0 to 3 V scan (Sweep 1). This transition from the OFF state to the ON state represents the information writing process. During the subsequent negative sweep from 0 to −3 V (Sweep 2), the device maintains its high‐conductivity state. The device remains in the ON state even under consecutive positive and negative voltage scans, indicating permanent retention of the written information. Thus, the device exhibits characteristics of a typical non‐volatile binary WORM memory with an I_ON_:I_OFF_ current ratio of 10^6^:1. The corresponding electrical performance is summarized in Table 1.

**TABLE 1:** The summary of electrical performance based all compounds.

Devices	NIZ‐methylene‐Azo	IMI‐methylene‐Azo	CZ‐methylene‐Azo	TPA‐methylene‐Azo
*V*_set_(V)	0.8, 1.8	0.8	0.5	1.0
*V_re_*_set_(V)	—	−2.3	−2.4	—
*I*_on_/*I*_off_	10^3^:10^2^:1	10:1	10^2^:1	10^6^:1

To evaluate the reliability and endurance of the devices based on NIZ‐, IMI‐, Cz‐, and TPA‐methylene‐Azo molecules, we investigated the stability of their high and low resistance states by measuring their retention characteristics. For the ternary NIZ‐methylene‐Azo device, the OFF, ON1, and ON2 states were read at 0.1, 1, and 2 V, respectively. For the binary devices, read voltages of 0.1 V (OFF) and 1 V (ON) were used for the IMI‐ and Cz‐based films, while 0.1 V (OFF) and 2 V (ON) were applied to the TPA‐based film. As shown in Figure 2f–i, all memory devices exhibited excellent stability with negligible current degradation over a testing period of 10^4^ s. These results indicate high endurance and a low probability of misreading. Figure S13a–d illustrates the cyclic endurance tests performed to assess the operational reliability of the devices. Throughout 100 consecutive resistive switching cycles, all devices maintained stable electrical characteristics without noticeable degradation, thereby verifying their high reproducibility. Additionally, the long‐term reliability was assessed by re‐testing the *I*–*V* curves after two months (Figure S14). The NIZ‐ and TPA‐based devices remained in the ON state, whereas the IMI‐ and Cz‐based devices continued to exhibit stable reversible (write/erase) switching. Collectively, these results underscore the superior stability and non‐volatile nature of memory devices.

To investigate the variation of threshold voltages, statistical analyses were performed on devices based on NIZ‐, IMI‐, Cz‐, and TPA‐methylene‐Azo. As shown in Figure S15a–d, all voltage distributions follow Gaussian behavior. For NIZ‐based devices, V_th1_ (1.05 ± 0.12 V) and V_th2_ (1.71 ± 0.13 V) exhibit narrow and similar distributions, indicating good uniformity and reproducibility. For IMI‐based devices, V_Set_ (0.67 ± 0.11 V) shows a more concentrated distribution than V_Reset_ (−2.04 ± 0.18 V), with only 24 out of 30 devices exhibiting a clear reset process, suggesting poorer reset stability. In contrast, Cz‐based devices show comparable distributions for V_Set_ (0.66 ± 0.14 V) and V_Reset_ (−2.45 ± 0.15 V), with 28 out of 30 devices displaying stable switching. Notably, TPA‐based devices exhibit the narrowest distribution V_th_ (1.10 ± 0.10 V), indicating superior uniformity and reproducibility.

Device yield is a key metric for evaluating the reliability of memory devices. An 8 × 8 array of NIZ‐methylene‐Azo, IMI‐methylene‐Azo, Cz‐methylene‐Azo, and TPA‐methylene‐Azo devices was randomly selected for statistical analysis. As summarized in Figure S16, 48, 25, 27, and 51 functional devices were obtained, corresponding to yields of 75%, 39.0%, 42.19%, and 76.56%, respectively. The higher yield of NIZ‐methylene‐Azo‐based devices is attributed to its superior solubility and smoother film morphology, which reduce interfacial Schottky barriers and facilitate stable carrier injection. In contrast, the lower yield of IMI‐methylene‐Azo devices likely arises from reduced effective solution concentration and increased interfacial inhomogeneity. Similarly, the relatively high yields observed for Cz‐methylene‐Azo and TPA‐methylene‐Azo devices can be correlated with their improved surface flatness, leading to enhanced device uniformity and reproducibility [33].

To verify the existence or not of the metal filaments arisen from the electrode materials, Au with high boiling point was used as the top electrode instead of Al to construct ITO/molecule/Au devices. As shown in Figure S17, the ITO/NIZ‐methylene‐Azo/Au and ITO/TPA‐methylene‐Azo/Au devices showed stable ternary and binary WORM behaviors, respectively, while the ITO/IMI‐methylene‐Azo/Au and ITO/Cz‐methylene‐Azo/Au devices exhibited bipolar nonvolatile resistive memory characteristics. The similarity of these results to those of the Al‐based devices confirms that the memory mechanism is intrinsic to the active layer and is not driven by the formation of metal conductive filaments.

**FIGURE 3:**
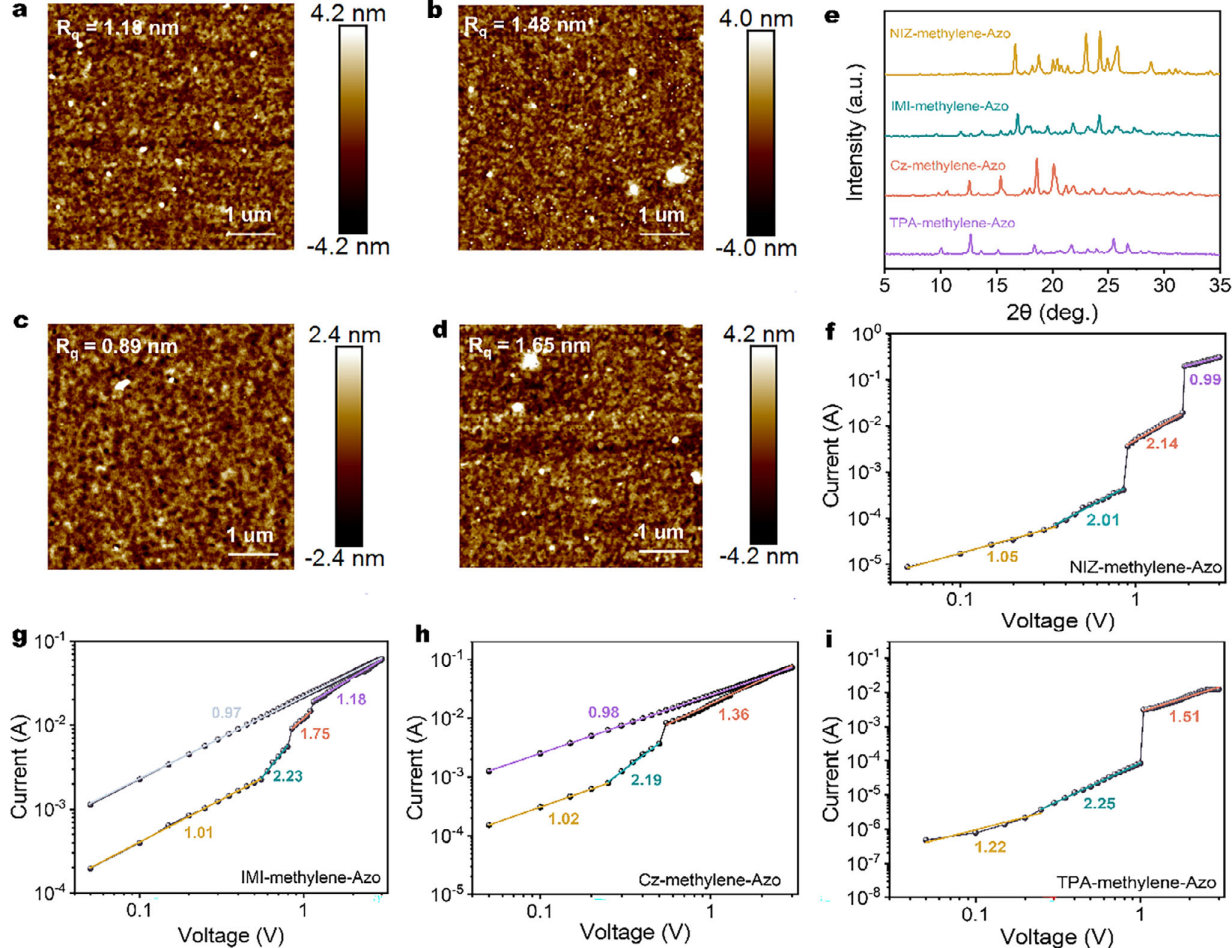
AFM height images of (a) NIZ‐methylene‐Azo, (d) IMI‐methylene‐Azo, (c) Cz‐methylene‐Azo and (d) TPA‐methylene‐Azo. (e) X‐ray diffraction patterns of NIZ‐methylene‐Azo, IMI‐methylene‐Azo, Cz‐methylene‐Azo and TPA‐methylene‐Azo thin films on quartz substrates. Image of the positive *I*–*V* curve plotted based on (f) NIZ‐methylene‐Azo, (g) IMI‐methylene‐Azo, (h) Cz‐methylene‐Azo and (i) TPA‐methylene‐Azo in the logarithmic scale and the corresponding fitting using a linear function for different portions.

To understand the impact of film microstructure on device performance, we analyzed the surface morphology of the films using atomic force microscopy (AFM). As shown in Figure 3a–d, the NIZ‐, IMI‐, Cz‐, and TPA‐methylene‐Azo films showed smooth surface morphologies with roughness values of 1.16, 1.48, 0.89, and 1.65 nm, respectively. Such smoothness guarantees robust electrode–film contact, thereby promoting carrier transport. Notably, the Cz‐methylene‐Azo device, which featured the minimum film roughness, exhibited the lowest V_th_. Subsequently, the crystallinity of the organic films was examined using x‐ray diffraction (XRD) technology. Figure 3e reveals that NIZ‐methylene‐Azo and IMI‐methylene‐Azo exhibit sharp diffraction peaks at 2θ = 28.8° and 27.3°, respectively. The corresponding small d‐spacings (3.09 and 3.26 Å) indicate highly ordered molecular packing, a key factor for efficient charge transport and high device performance. Similarly, TPA‐methylene‐Azo and Cz‐methylene‐Azo showed peaks at 26.7° (d = 3.34 Å) and 26.8 (d = 3.32 Å), respectively, which are characteristic of π–π stacking between conjugated molecular backbones. The relatively short *π*–*π* stacking distances indicate a compact molecular packing and enhanced intermolecular π‐orbital overlap, which is favorable for charge transport in organic semiconductors. Such ordered packing is expected to contribute to the improved electrical and synaptic performance of the corresponding devices. The superior crystallinity of Cz‐methylene‐Azo contributed to a higher quality film morphology and better carrier transport, resulting in the lowest V_th_ among the tested devices.

To elucidate the resistive switching mechanism, the *I*–*V* characteristics were analyzed using double‐logarithmic plots [34, 35, 36, 37]. As shown in (Figure 3f–i), all devices exhibit multiple linear regions with distinct slopes, indicating that different conduction mechanisms dominate the SET process at different bias ranges. The fitted slope (α) follows the power‐law relationship I ∝ V^α^, enabling clear identification of the transport regimes. In the low‐voltage OFF‐state region (0–0.4 V), the NIZ‐methylene‐Azo device exhibits Ohmic conduction with a slope of ∼1.05, attributed to thermally activated hole injection from the Al electrode into the HOMO level under a relatively high injection barrier [38, 39, 40], as shown in Figure 3e. Similar Ohmic behavior is observed by other devices at low bias. With increasing bias (0.5–0.85 V), the slope for the NIZ‐methylene‐Azo device increases to ∼2.01, indicating a transition to space‐charge‐limited current (SCLC) conduction [41, 42], as shown in Figure 3f. This behavior arises from enhanced hole injection due to Schottky barrier lowering, followed by progressive trap filling in the active layer. Likewise, the IMI‐methylene‐Azo device exhibits SCLC‐dominated transport in the 0.55–0.8 V range, where trapped carriers generate an internal electric field that facilitates further carrier trapping, as shown in Figure 3g. As the applied voltage approaches the threshold, the slopes for the Cz‐methylene‐Azo and TPA‐methylene‐Azo devices increase to 2.19 and 2.25, respectively, consistent with Child's law (I ∝ V^2^) and indicative of conductive pathway formation, as shown in Figure 3h,i. For the NIZ‐methylene‐Azo device, exceeding the first threshold voltage (∼0.85 V) leads to complete trap filling and the formation of a continuous conductive channel, accompanied by an SCLC slope of ∼2.14 during the transition to a high‐conductance state. Once the conductive channel is established, a sharp current increase marks the transition from the high‐resistance state (HRS) to the low‐resistance state (LRS). At higher bias (>1.85 V), the slope approaches ∼0.99, confirming Ohmic conduction in the LRS [43]. The IMI‐, Cz‐, and TPA‐methylene‐Azo devices exhibit similar Ohmic behavior in the LRS. Overall, the *I*–*V* characteristics of all devices are well described by the SCLC model, indicating that their memory behaviors are governed by trap‐assisted charge transport and CT processes.

**FIGURE 4:**
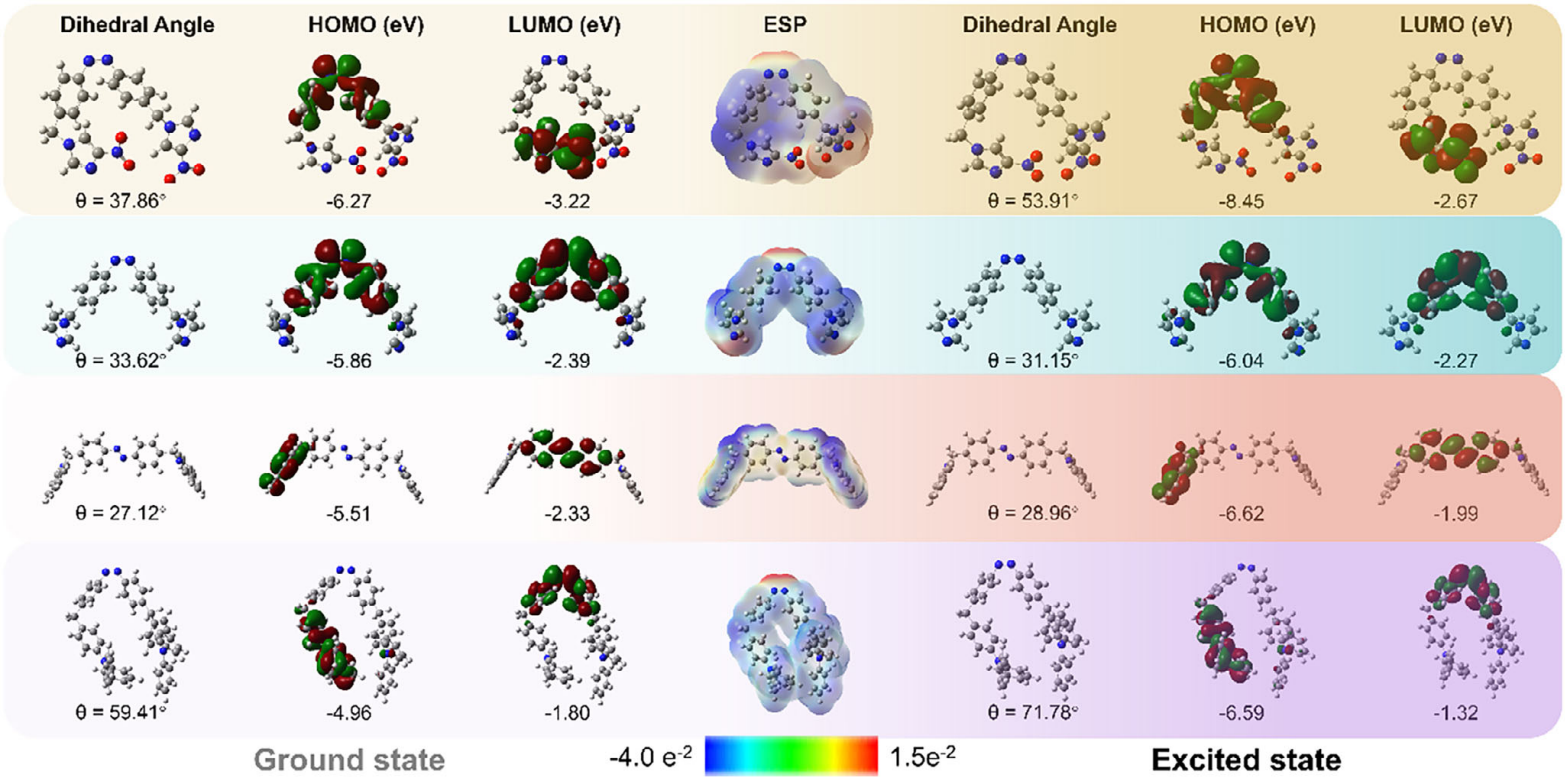
DFT results of NIZ‐, IMI‐, Cz‐, and TPA‐methylene‐Azo, including HOMO/LUMO distributions, dihedral angles (θ), and ESP.

To elucidate the interplay between electronic structure and memory characteristics, we employed molecular simulation and density functional theory (DFT) to calculate dihedral angles, frontier orbital energy levels (HOMO/LUMO), and electrostatic surface potentials (ESP) for both ground and excited states, as shown in Figure 4. The excellent agreement between calculated energy levels and experimental optical/electrochemical data substantiates the theoretical model.

The geometric configuration of all molecules simulated by theoretical calculation presents a wing‐like shape. Frontier orbital analysis indicates that the HOMO densities of NIZ‐ and IMI‐methylene‐Azo are predominantly localized on the azobenzene moiety. However, the strong electron‐withdrawing nitro group in NIZ‐methylene‐Azo localizes its LUMO density onto the nitroimidazole unit. In contrast, TPA‐ and Cz‐methylene‐Azo exhibit a distinct D‐A architecture, with HOMO densities centered on the triphenylamine or carbazole donors and LUMO densities on the azobenzene acceptor. ESP mapping reveals a continuous channel of negative potential (blue/white) along the molecular backbone, conducive to CT, while positive regions (red) associated with electron‐deficient groups act as carrier traps. Furthermore, the barrier for hole injection from ITO to the HOMO is found to be lower than that for electron injection from Al to the LUMO, favoring hole‐dominated transport. Under an applied external bias, carriers are injected and transported from the donor HOMO to the acceptor LUMO, leading to the formation of a charge‐separated state. As the bias approaches specific threshold voltages, trap sites—defined by the electron‐withdrawing strength of specific moieties—are sequentially filled, forming conductive filaments that switch the device state. For NIZ‐methylene‐Azo, shallow traps associated with the azobenzene group are filled first (“ON1” state), followed by the filling of deeper nitroimidazole traps at a higher threshold (“ON2” state). This trap‐filling process is accompanied by the formation of a conductive CT complex, evidenced by the significant deepening of the HOMO and elevation of the LUMO levels. While the azo moiety is inherently electronegative, the introduction of groups that enhance the electron cloud density on the backbone triggers a substantial torsion in the dihedral angle due to lone‐pair electrons repulsion. This modulation of the spatial conformation significantly impacts the electrical characteristics and performance of the device. This phenomenon aligns with the twisted intramolecular charge transfer (TICT) model, where electron transfer is coupled with a transition to a highly twisted conformation. Our methylene‐bridged design facilitates this conformation‐coupled CT. The resulting steric twisting creates an energy barrier that inhibits the dissociation of the CT complex, thereby stabilizing the conductive state. Specifically, NIZ‐methylene‐Azo undergoes a significant dihedral angle rotation (37.86° to 53.91°) upon excitation, securing a stable conductive path and resulting in non‐volatile ternary WORM behavior. Conversely, the IMI‐methylene‐Azo molecule lacks significant orbital separation or energy level shifts upon excitation. The absence of a strong push‐pull effect renders the charge‐separated state unstable, leading to easy erasure under reverse bias. For TPA‐methylene‐Azo, a substantial conformational distortion (59.41° to 71.78°) upon excitation, reinforced by the steric hindrance of the triphenylamine group, effectively locks the system in the conductive state, yielding non‐volatile binary WORM memory. While Cz‐methylene‐Azo undergoes a similar process, the conformational change is negligible (27.12° to 28.96°). Consequently, the excited state lacks the geometric stabilization required to prevent complex dissociation, leading to volatile, rewritable bipolar nonvolatile resistive memory characteristics.

**FIGURE 5:**
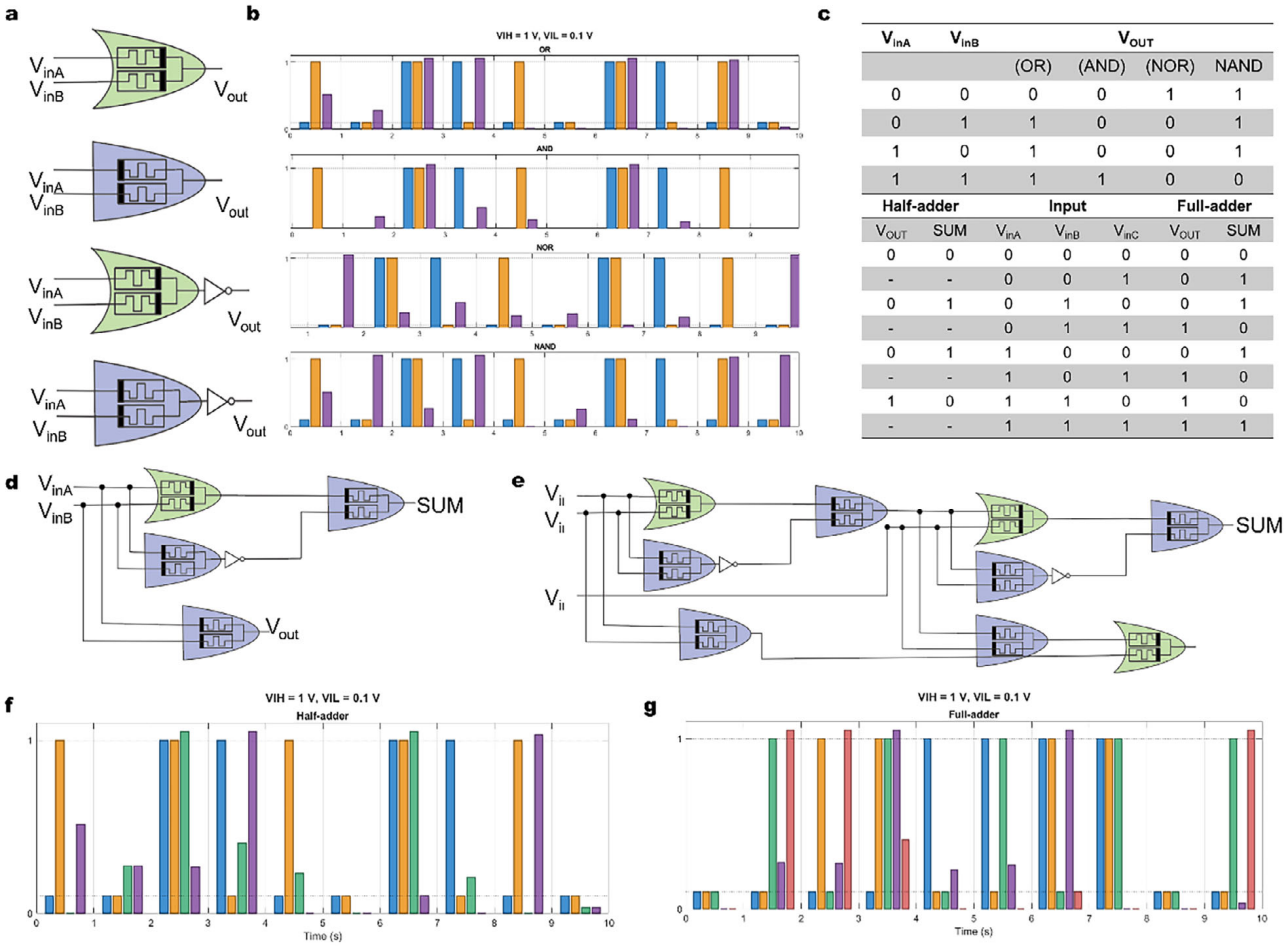
(a) Schematics of the Cz‐methylene‐Azo memristor‐based OR, AND, NOR and NAND logic gates. (b) Simulated output waveforms for the OR and AND logic operations. (c) Comprehensive truth tables for OR, AND, NOR, NAND, the half and full adders. Schematics of the (d) half adder and (e) full adder circuits utilizing memristors. Simulation outcomes for the (f) half adder and (g) full adder, respectively.

The voltage‐dependent conductance of the ITO/Cz‐methylene‐Azo/Al memristor makes it highly suitable for logic applications. By defining the device polarity such that forward current triggers the LRS and reverse current triggers the HRS, as shown in Figure 5a, we designed and simulated logic gates [44] in MATLAB Simulink. Simulation parameters (1 V/0.1 V inputs, 1 kΩ/7 kΩ resistances) were selected to match experimental data (Figure 2e). The realization of OR and AND gates rely on controlling the directionality of the memristors. Uniform positive orientation yields OR logic, while negative orientation yields AND logic. As evidenced by the waveforms in Figure 5b, the simulation outputs align perfectly with the theoretical truth tables (Figure 5c). To expand this library, CMOS inverters were introduced to create NOR and NAND gates (Figure 5a), demonstrating the device's compatibility with CMOS technology [45]. Furthermore, we integrated adder circuits by combining these logic gates [46, 47]. Figure 5d shows a half‐adder circuit that performs binary addition to generate SUM (XOR operation) and V_out_ (Carry/AND operation) signals. Simulations (Figure 5d) confirm the device's ability to execute half‐addition as per the truth table, as shown in Figure 5c. To address the carry‐bit limitation, a full adder was subsequently designed, as shown in Figure 5e. Verification against the truth table confirms that this memristor‐based full adder successfully handles three‐bit binary addition, as shown in Figure 5f,g. Additionally, XOR and XNOR logic gate circuits were also successfully designed and verified through simulation by combining OR, AND, and NAND logic gates in parallel configurations. The corresponding simulation outputs and truth tables are presented in Figure S18. Notably, these memristive logic architectures offer significant advantages over traditional CMOS circuits, including reduced transistor counts, lower power consumption, and higher integration density.

**FIGURE 6:**
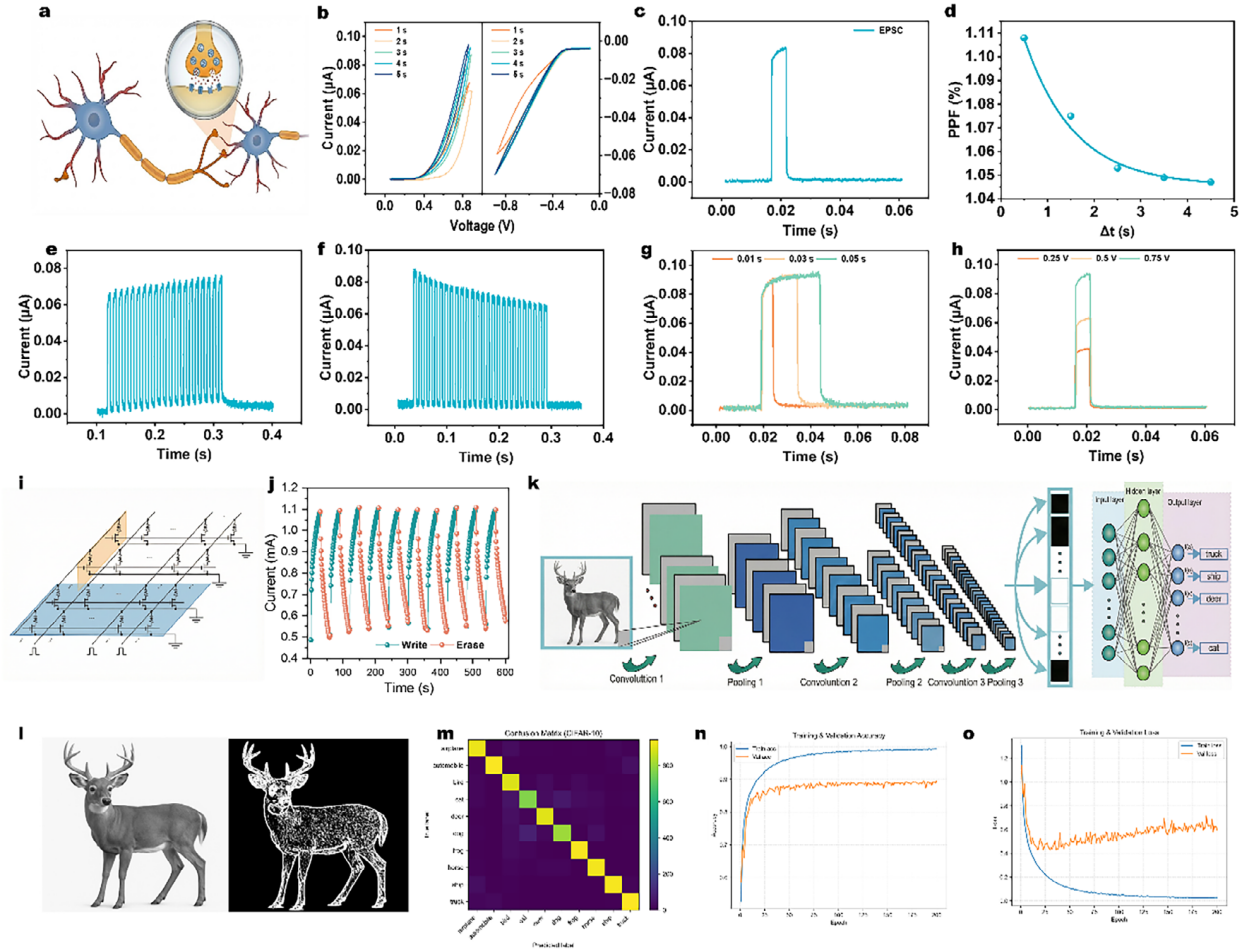
(a) Schematic of a biological synapse. (b) The I−V characteristics of the Cz‐methylene‐Azo‐based memristor were measured during positive (left) and negative (right) voltage sweeps. (c) EPSCs evoked by a single electric pulse (− 1 V, 0.02 s). (d) PPF index vs. pulse interval by applying a paired pulse. Current responses to 10 identical stimulation (e) positive voltage pulses (−1 V, 0.02 s) and (f) negative voltage pulses (+1 V, 0.02 s). (g) Current responses at various pulse voltage. (h) Current responses to presynaptic pulses with different pulse widths. (i) The crossbar with memristor. (j) Cyclic synaptic weight modulation (LTP/LTD) representing the learning and forgetting processes in the doped organic synapse. (k) Schematic diagram of the CNNs. (l) Input grayscale deer image and edge enhancement result after array processing. (m) Confusion matrix of the test set based on the memristive FC layer. Evolution of training/validation (n) accuracy and (o) loss over epochs.

In the central nervous system (CNS), neurons serve as the primary units for information processing. These cells establish connections and communicate through specialized junctions known as synapses. As illustrated in Figure 6a, a biological synapse typically comprises a presynaptic neuron, an intervening synaptic cleft, and a postsynaptic neuron. Figure 6b presents the representative I–V characteristics of the Cz‐methylene‐Azo‐based memristor measured over five consecutive voltage sweep cycles, including positive (0 → 1 → 0 V) and negative (0 → −1 → 0 V) scans at room temperature. Distinct pinched hysteresis loops are observed under bipolar periodic electrical stimulation, which is a hallmark characteristic of analogue memristive behavior. Figure 6c presents the EPSC characteristics of the Cz‐methylene‐Azo‐based memristor induced by an electrical presynaptic pulse (1 V, 20 ms). At room temperature, the EPSC showed a rapid rise upon electrical stimulation and then slowly decayed over time, resembling the excitatory synaptic behavior observed in biological systems. When two identical consecutive presynaptic voltage pulses were applied, the second EPSC peak (A2) was significantly higher than the first peak (A1), indicating the presence of PPF, as shown in Figure S19. This facilitation behavior originates from the incomplete relaxation of accumulated charge carriers induced by the first pulse, which enhances the conductance state and results in an increased EPSC upon the second stimulation. The PPF behavior was quantitatively evaluated using the PPF index, defined as A2/A1 × 100%. As the interval time (Δt) between the two presynaptic pulses increased from 0.5 to 4.5 s, the PPF index gradually decreased from 104.7% to 110.8%, following an exponential decay trend, as shown in Figure 6d. This behavior closely resembles the temporal learning and memory characteristics of biological synapses, where the synaptic facilitation effect weakens as the interval between stimuli increases.

Notably, when a series of consecutive presynaptic pulses were delivered to the the Cz‐methylene‐Azo‐based memristor, the device exhibited typical short‐term plasticity characteristics, including progressive potentiation of the postsynaptic current and, under alternative stimulation conditions, a gradual depression behavior, as shown in Figure 6e,f. The synaptic characteristics of the device were further modulated by the electrical pulse conditions. As illustrated in Figure 6g, increasing the pulse width led to more pronounced synaptic responses, demonstrating enhanced conductance modulation. Likewise, higher pulse amplitudes induced larger postsynaptic currents (Figure 6h), indicating the potential of the device for the application in neuromorphic computing.

With the rapid development of IoT, edge computing has become essential for efficient local data processing. Memristor‐based in‐sensor computing enables fast and energy‐efficient edge intelligence by integrating sensing and computing within a single platform [48]. Figure 6i illustrates the mapping of the Sobel kernel onto the crossbar, where pixel voltages are applied to rows to compute local gradients via column current summation based on Kirchhoff's law. Figure 6j demonstrates the emulation of variable synaptic weights through continuous conductance tuning. By applying a train of 25 negative pulses (−1 V, 20 ms) followed by 25 positive pulses (+1 V, 20 ms), the device achieved LTP and LTD, respectively. A 0.1 V read bias, insufficient to alter the device state, was used to track the conductance after each pulse. Moreover, experimental trials on random devices revealed minimal variation in output current during repeated LTP/LTD sequences. Such findings demonstrate the excellent reproducibility of the devices and confirm that stable, repeatable electrically modulation of synaptic plasticity is achievable. Building on these characteristics, we constructed a natural image recognition framework, as shown in Figure 6k. The system integrates a memristive edge‐detection frontend with a hybrid “CNN + memristor” architecture, known for high energy efficiency [49, 50, 51]. To accentuate object contours and reduce redundancy, a memristive edge enhancement module precedes the CNN. Figure 6l shows the original grayscale input, and the processed image exhibits enhanced contours with effectively suppressed background noise. The CNN architecture extracts hierarchical features via three convolution/pooling layers, which are compressed into 256‐dimensional vectors via global average pooling. The final fully connected (FC) layer is physically implemented by the memristive crossbar for classification. The network was evaluated on the CIFAR‐10 benchmark [52]. After training with standard augmentation and the Adam optimizer, the floating‐point FC weights were quantized and mapped to the crossbar to perform inference via analog vector‐matrix multiplication (VMM) [53]. The confusion matrix (Figure 6m) shows high recognition accuracy for structurally distinct classes (e.g., airplanes, automobiles), with expected confusion among texturally similar classes (e.g., cats/dogs), consistent with software baselines. Training dynamics show validation accuracy saturating at ∼87% after 40 epochs, as shown in Figure 6n,o. Notably, incorporating quantization and moderate device variability caused only negligible performance degradation, confirming the robustness of the mapping strategy. To further verify the universality and robustness of the proposed memristive edge‐computing paradigm, we extended the evaluation to a broader range of randomly selected images from the CIFAR‐10 dataset. As illustrated in Figure S20, ten representative samples were tested, covering distinct categories including man‐made objects (airplane, truck, automobile, ship) and animals (cat, bird, frog, horse). The results demonstrate that the Cz‐methylene‐Azo memristor crossbar consistently performs high‐quality analog convolution across diverse subjects. It is evident that the device array effectively differentiates between high‐frequency contour information and low‐frequency background noise. Whether processing the rigid, linear boundaries of mechanical structures (e.g., the fuselage of the airplane in Figure S20a and the hull of the ship in Figure S20d) or the complex, irregular textures of biological entities (e.g., the fur of the cat in Figure S20e), the system preserves critical shape features with high fidelity. This demonstrates that the proposed memristive crossbars can synergistically perform analog edge enhancement and classification. The system achieves near‐software accuracy, offering a viable, energy‐efficient solution for hardware‐implemented edge intelligence.

## 3 Conclusions

In summary, we have designed and synthesized a series of wing‐shaped symmetric azobenzene‐based small molecules with tunable D‐A characteristics by introducing different terminal substituents via methylene linkers. Systematic investigations reveal that the strength and number of electron‐withdrawing groups, together with the charge separation state and conformationally coupled CT process, play decisive roles in determining the resistive switching behavior of the corresponding memory devices. By molecular engineering, diverse memory characteristics—including nonvolatile ternary or binanry WORM, and bipolar nonvolatile resistive memory behaviors—were successfully achieved within a unified molecular framework. Moreover, the devices exhibit diverse electrically tunable synaptic functionalities, including multilevel conductance modulation, EPSC, PPF, and long‐term potentiation and depression, highlighting their strong potential for neuromorphic computing applications. Mechanism analyses indicate that CT induced molecular conformational changes introduce distinct transition‐state energy barriers, leading to different dissociation degrees of CT complexes and thus multiple stable or metastable resistance states. Beyond memory functionality, the devices were further demonstrated as logic‐in‐memory units capable of implementing various logic gates, as well as half‐adder and full‐adder circuits. Their potential for in‐memory computing was further validated through CNN–based image edge detection tasks. These results highlight the effectiveness of rational molecular design in regulating resistive switching and demonstrate the potential of azobenzene‐based organic molecular systems for multifunctional memory and integrated computing applications.

## 4 Experimental Section

### 4.1 Synthesis of Me‐Azo

Cleaned magnesium turnings (5.79 g, 0.24 mol) were placed in a 250 mL three‐necked flask. Subsequently, a solution of *p*‐nitrotoluene (6.52 g, 0.048 mol) and 150 mL of methanol was added. The mixture was stirred at room temperature for 2 h. The reaction mixture was neutralized to a weakly acidic pH using dilute hydrochloric acid, followed by adjustment to a neutral pH with aqueous NaHCO_3_. The precipitate was collected by filtration, washed, and dried. The crude product was purified by column chromatography on silica gel (eluent: petroleum ether) to yield a yellow solid (2 g, 40% yield). ^1^H NMR (400 MHz, Chloroform‐*d*) δ 7.87–7.76 (m, 4H), 7.30 (d, *J* = 7.8 Hz, 4H), 2.43 (s, 6H).

### 4.2 Synthesis of Br‐Methylene‐Azo

To a 100 mL three‐necked flask were added Me‐Azo (300 mg, 1.43 mmol), benzoyl peroxide (BPO, 20 mg, 0.08 mmol), and N‐bromosuccinimide (NBS, 760 mg, 3 mmol). Chloroform (50 mL) was added under a nitrogen atmosphere. The system was degassed and purged with nitrogen three times, then heated at 65°C for 18 h. After cooling to room temperature, the reaction mixture was filtered. The orange filter cake was washed sequentially with water and dichloromethane, then dried under vacuum to afford an orange‐yellow solid (274 mg, 52% yield). ^1^H NMR (600 MHz, Chloroform‐*d*) δ 7.89 (d, *J* = 8.3 Hz, 4H), 7.54 (d, *J* = 8.3 Hz, 4H), 7.31 – 7.28 (m, 1H), 6.87 – 6.81 (m, 1H), 4.56 (s, 4H), 4.42 (s, 1H).

### 4.3 Synthesis of NIZ‐Methylene‐Azo

4‐Nitroimidazole (271 mg, 2.4 mmol) and sodium hydride (72 mg, 3 mmol) were added to a reaction flask. Anhydrous THF (30 mL) was added under a nitrogen atmosphere, and the mixture was stirred at room temperature for 2 h. Subsequently, Br‐methylene‐Azo (368 mg, 1 mmol) was added, and the mixture was heated with stirring for 18 h. After cooling to room temperature, the mixture was poured into water and extracted with dichloromethane. The organic layer was dried over anhydrous MgSO_4_, filtered, and concentrated under reduced pressure. The residue was purified by column chromatography (eluent: dichloromethane/methanol = 50:1) to yield a yellow solid (258 mg, 70% yield). ^1^H NMR (400 MHz, DMSO‐*d*6) δ 8.55 (d, *J* = 1.5 Hz, 2H), 8.06 (d, *J* = 1.5 Hz, 2H), 7.91 (d, *J* = 8.2 Hz, 4H), 7.63 – 7.54 (m, 4H), 5.44 (s, 4H). ^13^C NMR (101 MHz, DMSO‐*d*6) δ 152.10, 147.73, 140.15, 138.06, 129.54, 123.64, 122.17, 50.79. LC‐MS: m/z calcd for: 432.14, found: 432.2[M + H]^+^.

### 4.4 Synthesis of IMI‐Methylene‐Azo

To a 100 mL reaction flask were added imidazole (164 mg, 2.4 mmol) and sodium hydride (72 mg, 3 mmol). Anhydrous THF (30 mL) was added under a nitrogen atmosphere, and the mixture was stirred at room temperature for 2 h. Br‐methylene‐Azo (368 mg, 1 mmol) was then added, and the mixture was heated with stirring for 18 h. Upon completion, the reaction mixture was cooled to room temperature, poured into water, and extracted with dichloromethane. The organic phase was dried over anhydrous MgSO_4_, filtered, and concentrated under reduced pressure. The crude product was purified by column chromatography (eluent: dichloromethane/methanol = 50:1) to obtain a yellow solid (240 mg, 70% yield). ^1^H NMR (600 MHz, Chloroform‐*d*) δ 7.90 (d, *J* = 8.1 Hz, 4H), 7.60 (s, 2H), 7.29 (d, *J* = 8.1 Hz, 4H), 7.13 (s, 2H), 6.94 (s, 2H), 5.21 (s, 4H).^13^C NMR (101 MHz, Chloroform‐*d*) δ 152.43, 139.30, 137.63, 130.18, 128.09, 123.66, 119.46, 50.58. LC‐MS: m/z calcd for: 342.41, found: 343.3[M + H]^+^.

### 4.5 Synthesis of Cz‐Methylene‐Azo

Br‐methylene‐Azo (368 mg, 1 mmol), carbazole (417.5 mg, 2.5 mmol), and potassium hydroxide (420 mg, 7.5 mmol) were placed in a 100 mL reaction flask. After multiple vacuum/nitrogen cycles, anhydrous toluene (60 mL) was added. The mixture was heated at 110°C for 12 h. After cooling, the solution was poured into deionized water and extracted with dichloromethane. The organic layer was dried over anhydrous MgSO_4_, filtered, and concentrated under reduced pressure. The crude product was purified by column chromatography (eluent: petroleum ether/dichloromethane = 1:2) to yield a yellow solid (231 mg, 42.6% yield). ^1^H NMR (400 MHz, Chloroform‐d) δ 8.07 (d, *J* = 7.7 Hz, 4H), 7.69 (d, *J* = 8.5 Hz, 4H), 7.36 (ddd, *J* = 8.3, 7.1, 1.2 Hz, 4H), 7.29 (d, *J* = 8.2 Hz, 4H), 7.21 (d, *J* = 1.1 Hz, 4H), 7.18 – 7.15 (m, 4H), 5.51 (s, 4H).^13^C NMR (101 MHz, Chloroform‐d) δ 140.69, 140.36, 127.24, 126.08, 123.43, 123.24, 120.61, 119.53, 108.96, 46.52, 0.15. LC‐MS: m/z calcd for: 540.67, found: 541.3.[M + H]^+^.

### 4.6 Synthesis of TPA‐Methylene‐Azo

To a 100 mL reaction flask were added Br‐methylene‐Azo (368 mg, 1 mmol), 4‐(4,4,5,5‐tetramethyl‐1,3,2‐dioxaborolan‐2‐yl)triphenylamine (triphenylamine‐4‐boronic acid pinacol ester, 816.82 mg, 2.2 mmol), and potassium carbonate (138 mg, 1 mol). The flask was purged with nitrogen once. Pd(PPh_3_)_4_ (25 mg, 0.005 mmol), toluene (12 mL), deionized water (8 mL), and ethanol (4 mL) were added. The system was degassed and purged with nitrogen three times. The mixture was stirred and slowly heated to 95°C, then maintained at this temperature for 24 h. After cooling to room temperature, the mixture was poured into water and extracted with dichloromethane. The organic layer was dried over anhydrous MgSO_4_, filtered, and concentrated. The residue was purified by column chromatography (eluent: petroleum ether/dichloromethane = 3:1) to yield a yellow solid (321 mg, 46% yield). ^1^H NMR (400 MHz, DMSO‐*d_6_*) δ 7.82 (d, *J* = 6.5 Hz, 4H), 7.47 (d, *J* = 8.4 Hz, 4H), 7.29 – 7.25 (m, 8H), 7.21 (d, *J* = 8.5 Hz, 4H), 7.01 (d, *J* = 7.3 Hz, 4H), 6.96 (dd, *J* = 8.0, 4.0 Hz, 12H), 4.00 (s, 4H). ^13^C NMR (101 MHz, Chloroform‐d) δ 151.40, 147.99, 146.21, 144.59, 135.09, 129.84, 129.76, 129.31, 124.59, 124.12, 123.10, 122.68, 109.80, 41.36, 0.15. LC‐MS: m/z calcd for: 694.92, found: 696.3.[M + H]^+^.

### 4.7 Device Fabrication and Characterization

The memory devices were fabricated with a conventional sandwich‐like configuration of ITO/Active Layer/Al. ITO glass substrate were cleaned sequentially by ultrasonic agitation in acetone, deionized water, and isopropanol for 25 min each. The cleaned substrates were then subjected to UV‐ozone treatment for 20 min to enhance interfacial compatibility. Subsequently, the active layer films were deposited onto the ITO substrates by spin‐coating THF solutions of NIZ‐methylene‐Azo, IMI‐methylene‐Azo, Cz‐methylene‐Azo, and TPA‐methylene‐Azo (20 mg/mL) at an optimized speed of 3000 rpm. The films were then annealed at 80°C for 25 min. Finally, top aluminum (Al) or Au electrodes (150 nm) were deposited through a shadow mask (diameter = 300 µm) via thermal evaporation at a base pressure of 2.5 × 10^− 6^ Torr. The electrical current‐voltage (*I‐V*) characteristics of the devices were measured using a Keithley 4200‐SCS semiconductor characterization system under ambient light conditions.

## Conflicts of Interest

The authors declare no conflict of interest.

## Supporting information




**Supporting File**: advs76309‐sup‐0001‐SuppMat.

## Data Availability

The data that support the findings of this study are available from the corresponding author upon reasonable request.
