# Supplementary material for: Molecularly Engineered Wing‐Shaped Azobenzene Memristors for Logic‐in‐Memory and Edge Visual Intelligence
Source: Adv Sci (Weinh). 2026 Jun 26;13(53):e76309. doi: 10.1002/advs.76309 (PMC13336838; doi:10.1002/advs.76309)
Supplement: Supplementary file 1 [file ADVS-13-e76309-s001.docx]

**Molecularly Engineered Wing-Shaped Azobenzene Memristors for Logic-in-Memory and Edge Visual Intelligence**

Yanze Liu,^1,3^ Tao Han^2*^, Jiahui Ding,^1,3^ Hong Lian,^1,3,^^4*^ Lingling Yao,^1,3^ Zhaoxin Xu,^1,3^ Xingyu Zhang,^1,3^ Shuanglong Wang,^4^ Jiangnan Xia,^2,4^ Tianchi Zhang,^4^ Weiwei Kang^5*^ and Qingchen Dong^1,3*^

^1^MOE Key Laboratory of Advanced Display and System Applications, Shanghai University, No.149 Yanchang Road, Jingan District, Shanghai 200072, China.

^2^Microelectronics and Optoelectronics Technology Key Laboratory of Hunan Higher Education, School of Physics and Electronic Electrical Engineering, Xiangnan University, Chenzhou 423000, P. R. China.

^3^School of Mechanical & Electronic Engineering and Automation, Shanghai University, No.99 Shangda Road, Baoshan District, Shanghai 200444, China.

^4^Department of Applied Physics, The Hong Kong Polytechnic University, Hong Kong SAR 999077, P.R. China.

^5^Third Hospital of Shanxi Medical University, Shanxi Bethune Hospital, Shanxi Academy of Medical Sciences, Tongji Shanxi Hospital, Taiyuan 030032, China.

*Correspondence: [than@xnu.edu.cn](mailto:than@xnu.edu.cn); hlian@polyu.edu.hk; kangweiwei@sxmu.edu.cn; [qcdong@shu.edu.cn](mailto:qcdong@shu.edu.cn)

**Scheme S1.** Synthetic routes of NIZ-methylene-Azo, IMI-methylene-Azo, Cz-methylene-Azo, and TPA-methylene-Azo.

**Fig.S1.** ^1^H NMR spectrum of NIZ-methylene-Azo in DMSO-d6.

**Fig. S2.** ^1^H NMR spectrum of IMI-methylene-Azo in CDCl_3_.

**Fig. S3.** ^1^H NMR spectrum of Cz-methylene-Azo in CDCl_3_.

**Fig. S4.** ^1^H NMR spectrum of TPA-methylene-Azo in DMSO-d6.

**Fig. S5.** ^13^C NMR spectrum of NIZ-methylene-Azo in DMSO-d6.

**Fig. S6.** ^13^C NMR spectrum of IMI-methylene-Azo in CDCl_3_.

**Fig. S7.** ^13^C NMR spectrum of CZ-methylene-Azo in CDCl_3_.

**Fig. S8.** ^13^C NMR spectrum of TPA-methylene-Azo in CDCl_3_.

**Fig. S9.** HRMS spectrum of NIZ-methylene-Azo.

**Fig. S10.** HRMS spectrum of IMI-methylene-Azo.

**Fig. S11.** HRMS spectrum of CZ-methylene-Azo.

**Fig. S12.** HRMS spectrum of TPA-methylene-Azo.

**Fig. S13.** Cycle tests of memory device for (a) NIZ-methylene-Azo, (a) IMI-methylene-Azo, (c) Cz-methylene-Azo and (d) TPA-methylene-Azo.

**Fig. S14.** *I–V* curves of (a) NIZ-methylene-Azo, (b) IMI-methylene-Azo, Cz-methylene-Azo, (d) and TPA-methylene-Azo after a period of two months.

**Fig. S15.** The distribution histogram and Gaussian fitting curves of the V_th_ values of (g) NIZ-methylene-Azo, (h) IMI-methylene-Azo and (i) Cz-methylene-Azo based devices.

**Fig. S16.** The statistical data of reproducibility of the devices based on (a) NIZ-methylene-Azo, (b) IMI-methylene-Azo, (c) Cz-methylene-Azo and (d) TPA-methylene-Azo.

**Fig. S17.** The I–V characteristic of memory device ITO/molecule/Au, (a) NIZ-methylene-Azo, (b) IMI-methylene-Azo, (c) Cz-methylene-Azo and (d) TPA-methylene-Azo, respectively.

**Fig. S18.** Cz-methylene-Azo-based implementations of logic gate circuits: (a) XOR gate and (b) XNOR gate. (c) Corresponding truth tables for the XOR and XNOR operations. (d) Simulated circuit responses of the XOR and XNOR logic gates.

**Fig. S19.** EPSCs elicited by a pair of electric pulses (− 1 V, 0.02 s) with an interval time of 0.02 s.

**Fig. S20.** Demonstration of image edge detection results using memristor-based Sobel computation. The images are arranged in three columns: original image, memristor Sobel gradient result, and memristor Sobel binarization result. Each row corresponds to a different recognition object: (a) airplane, (b) truck, (c) car, (d) ship, (e) cat, (f) bird, (g) frog, (h) horse.

**Table S1.** The photophysical properties for NIZ-methylene-Azo, IMI-methylene-Azo, Cz-methylene-Azo and TPA-methylene-Azo.

| **Molecules** | | **NIZ-methylene-Azo** | **IMI-methylene-Azo** | **Cz-methylene-Azo** | **TPA-methylene-Azo** |
| --- | --- | --- | --- | --- | --- |
| **λ_abs_ (nm)** | **Solution** | 331/443 | 328/443 | 293/328/342/440 | 304/457 |
|  | **Film** | 333 | 329 | 296/334/345 | 308 |

**Table S2.** Photophysical and electrochemical properties of NIZ-methylene-Azo、 IMI-methylene-Azo、 Cz-methylene-Azo and TPA-methylene-Azo.
